# Effect of adiponectin on bovine granulosa cell steroidogenesis, oocyte maturation and embryo development

**DOI:** 10.1186/1477-7827-8-23

**Published:** 2010-03-10

**Authors:** Virginie Maillard, Svetlana Uzbekova, Florence Guignot, Christine Perreau, Christelle Ramé, Stéphanie Coyral-Castel, Joëlle Dupont

**Affiliations:** 1 Unité de Physiologie de la Reproduction et des Comportements, UMR85, Equipe Métabolisme et Reproduction, INRA, F-37 380 Nouzilly, France

## Abstract

**Background:**

Adiponectin is an adipokine, mainly produced by adipose tissue. It regulates several reproductive processes. The protein expression of the adiponectin system (adiponectin, its receptors, AdipoR1 and AdipoR2 and the APPL1 adaptor) in bovine ovary and its role on ovarian cells and embryo, remain however to be determined.

**Methods:**

Here, we identified the adiponectin system in bovine ovarian cells and embryo using RT-PCR, immunoblotting and immunohistochemistry. Furthermore, we investigated in vitro the effects of recombinant human adiponectin (10 micro g/mL) on proliferation of granulosa cells (GC) measured by [3H] thymidine incorporation, progesterone and estradiol secretions measured by radioimmunoassay in the culture medium of GC, nuclear oocyte maturation and early embryo development.

**Results:**

We show that the mRNAs and proteins for the adiponectin system are present in bovine ovary (small and large follicles and corpus luteum) and embryo. Adiponectin, AdipoR1 and AdipoR2 were more precisely localized in oocyte, GC and theca cells. Adiponectin increased IGF-1 10(-8) M-induced GC proliferation (P < 0.01) but not basal or insulin 10(-8) M-induced proliferation. Additionally, adiponectin decreased insulin 10(-8) M-induced, but not basal or IGF-1 10(-8) M-induced secretions of progesterone (P < 0.01) and estradiol (P < 0.05) by GC. This decrease in insulin-induced steroidogenesis was associated with a decrease in ERK1/2 MAPK phosphorylation in GC pre-treated with adiponectin. Finally, addition of adiponectin during in vitro maturation affected neither the percentage of oocyte in metaphase-II nor 48-h cleavage and blastocyst day 8 rates.

**Conclusions:**

In bovine species, adiponectin decreased insulin-induced steroidogenesis and increased IGF-1-induced proliferation of cultured GC through a potential involvement of ERK1/2 MAPK pathway, whereas it did not modify oocyte maturation and embryo development in vitro.

## Background

Adiponectin is a hormone mainly produced by mature adipocytes and abundantly present in the circulation (2 to 20 μg/mL) in mammals [1,2]. This adipokine, also known as Acrp30, AdipoQ and GBP28, is a 30 kDa glycoprotein present in plasma as homomultimers, including trimers, hexamers and high molecular weight multimers [3]. Adiponectin plays an important role in the control of lipid metabolism, glucose homeostasis and insulin sensitivity [3,4]. Moreover, several studies in humans have shown that low circulating levels of adiponectin are associated with obesity [1,5], Type 2 diabetes [6] and metabolic syndrome [7], suggesting a strong link between adiponectin and insulin resistance [3]. Metabolic abnormalities can lead to development of some physiopathological situations affecting reproduction, such as the polycystic ovary syndrome (PCOS). This metabolic disorder is the most common cause of anovulation and infertility in reproductive-age women [8]. It is associated with hyperinsulinemia, hyperandrogenism and insulin resistance [9]. Low levels of circulating adiponectin have been found in both lean and obese women with PCOS compared to non-PCOS counterparts [10]. Recently, some evidence suggests that adiponectin could be involved in the control of reproductive functions [4,11]. Indeed, overexpression of circulating adiponectin impairs female fertility in mice [12], whereas the absence of adiponectin has no effect [13,14]. Additionally, our team has found in rat [2], chicken [15] and human [16], that physiological levels of recombinant human adiponectin (rh adiponectin, 5 or 10 μg/ml) are able to increase progesterone and/or estradiol secretions in response to insulin-like growth factor-I (IGF-I) in cultured granulosa cells.

Adiponectin exerts its action by binding at least to two seven-transmembrane domain-containing specific receptors, Adiponectin Receptors 1 and 2 (AdipoR1 and AdipoR2) [17]. These receptors belong to a newly described family of receptors, the progestin and adipoQ receptors (PAQR) [18], which are distinguishable from G protein-coupled receptors (GPCR) notably by their cytoplasmic N-terminus and extracellular C-terminus extremities [17,18]. These two receptors become biologically active by binding to an adaptor protein, containing a pleckstrin homology domain, a phosphotyrosine-binding domain and a leucine zipper motif (APPL1) [19]. They activate various intracellular signaling pathways including AMP-activated protein kinase (AMPK), peroxisome proliferator-activated receptor-α (PPARα), protein kinase B (Akt), p38 and p44/42 (ERK_1/2_) mitogen activated protein kinase (MAPK)], which are implicated in the regulation of energy metabolism [17,19,20]. Recently, AdipoR1 and AdipoR2 expression has been reported in ovary in human [16], pig [21], rodents [2], chicken [15] and cow [22]. Other reproductive organs express these two receptors as well, including hypothalamus [23] pituitary [23], oviduct [24], endometrium [25] and placenta [26]. All these data suggest that adiponectin could directly act on reproductive axis in different species.

A great decline of fertility has been observed worldwide in high yielding dairy cows since the last few decades [27]. In early lactation, high yielding dairy cows are under important metabolic stress, as they cannot meet the energetic demands for milk production entirely from feed intake [28]. To compensate for this energy deficit, the high yielding dairy cows greatly mobilize their energy stores mainly from their adipose tissue [29]. The negative energy balance, accompanied by several metabolic changes (e.g. hypoglycemia, hypoinsulinemia, decreased insulin sensitivity and high circulating levels of non-esterified fatty acids), could affect fertility by increasing the duration of the anestrus period and decreasing quality of oocytes and embryo [30,31]. Some evidence suggests the involvement of adiponectin in bovine reproduction [22,32]. The presence of AdipoR1 and AdipoR2 messenger RNA (mRNA) has been shown in bovine theca and granulosa cells from small and large follicles [22,32]. Moreover, recombinant human adiponectin decreases LH and insulin-induced progesterone and androstenedione secretions by bovine theca cells from large follicles [22]. Thus, adiponectin may act on bovine steroidogenesis in theca cells, but the protein expression of the entire adiponectin system in ovarian cells and the role of adiponectin in granulosa cells and oocyte and embryo quality remain to be studied in this species.

The objective of this work was first to investigate the mRNA and protein expression of the adiponectin system (adiponectin, AdipoR1, AdipoR2 and APPL1) in bovine ovarian cells and embryos. We also studied the effects of rh adiponectin on proliferation, steroidogenesis and different signaling pathways (ERK_1/2 _and p38 MAPK, Akt and AMPK) in bovine granulosa cells (GC) from small follicles and on bovine oocyte maturation and early embryo development *in vitro*.

## Methods

### Hormones, antibodies and chemicals

Recombinant human (rh) adiponectin produced in eukaryotic cells was purchased from Biovendor Research and Diagnostic Products (Heidelberg, Germany) and rh IGF-I and rh insulin were obtained from Sigma-Aldrich (Lyon, France).

Rabbit polyclonal antibodies to human APPL1, Akt, Phospho-AMPKα (Thr172), AMPKα, Phospho-p44/42 MAPK (ERK_1/2_) (Thr202/Tyr204), Phospho-p38 MAP Kinase (Thr180/Tyr182) and rabbit monoclonal antibodies to human Adiponectin (C45B10) were from Cell Signaling Technology (Ozyme, Saint Quentin Yvelines, France). Rabbit polyclonal antibodies to phospho-Akt1/2/3 (Ser473)-R, ERK_2 _(C-14), p38α (C-20) and Acrp30 (N-20)-R were obtained from Santa Cruz Biotechnology (Euromedex, Souffelweyersheim, France). Mouse monoclonal antibodies to Vinculin (clone hVIN-1) were purchased from Sigma-Aldrich. Rabbit polyclonal antibodies to human adiponectin Receptor1, human adiponectin Receptor 2 and goat polyclonal antibodies to human, mouse and rat adiponectin Receptor 2 were obtained from Immuno-Biological Laboratories International (Hamburg, Germany) and Novus Biologicals (Cambridge, United Kingdom), respectively. Horseradish peroxidase-conjugated anti-rabbit, anti-mouse IgG and anti-goat IgG were purchased from Eurobio (Les Ulis, France). Unless otherwise stated in the text, chemicals were obtained from Sigma-Aldrich.

### Tissue samples, isolation and culture of granulosa cells and cumulus-oocyte complexes (COC)

All procedures were approved by the Agricultural Agency and the Scientific Research Agency and conducted in accordance with the guidelines for Care and Use of Agricultural Animals in Agricultural Research and Teaching.

Bovine tissues were collected at the slaughterhouse. Cow ovaries and abdominal adipose tissue were frozen in liquid nitrogen and stored at -80°C for mRNA and protein characterization. Bovine ovaries, used for cell cultures, were transferred to physiological serum until the dissection.

Granulosa cells were isolated by puncturing small follicles (< 6 mm) allowing the expulsion of cells in the culture medium. The composition of this medium, called enriched McCoy's 5A, was as follow: McCoy's 5A, L-glutamine (3 mmol/L), hepes (20 mmol/L, pH = 7.6), Bovine Serum Albumin (BSA, 0.1%; Euromedex), penicillin (100 × 10^3 ^UI/L; Eurobio, Les Ulis, France), streptomycin (100 mg/L; Eurobio), bovine apo-transferrin (5 mg/L), selenium (0.25 μmol/L) and androstenedione (0.1 μmol/L). Cells were recovered by centrifugation, washed with fresh medium and counted in a haemocytometer. Cells were initially cultured for 24 h in enriched McCoy's 5A medium supplemented with 10% fetal bovine serum (FBS, PAA laboratories, Les Mureaux, France) and amphotericin B (2.7 μmol/L; Eurobio) without treatment and then incubated in fresh culture medium with or without test reagents for the appropriate time. Cultures were performed at 37°C under a humidified atmosphere with 5% CO_2 _in 95% air.

COC were obtained by aspiration of 3-6 mm antral follicles. Only COC with several layers of compact cumulus cells surrounding the oocyte were selected and washed several times in Tissue Culture Medium 199 (TCM 199)/Hepes medium supplemented with gentamycin (50 mg/L). *In vitro *maturation of COC (50 COC/500 μL) was performed in TCM 199 or in TCM 199 mix serum-free media with or without test reagents for 24 h at 38.8°C in water-saturated atmosphere of 5% CO_2 _in air. The TCM 199 mix medium was composed of TCM 199, EGF (10 ng/mL), IGF-I (19 ng/mL), FGF (2.2 ng/mL), hCG (5 IU/mL), PMSG (10 IU/mL), insulin (5 μg/mL), transferrin (5 μg/mL), selenium (5 ng/mL), L-cystein (90 μg/mL), β-mercaptoethanol (0.1 mM), ascorbic acid (75 μg/mL), glycine (720 μg/mL), glutamine (0.1 mg/mL) and pyruvate (110 μg/mL) [33].

### In vitro fertilization (IVF) and in vitro development (IVD)

After 24 h of IVM in TCM 199 mix or TCM 199 serum-free medium supplemented with or without adiponectin (10 μg/mL) and/or insulin (10^-8 ^M) and/or EGF (10 ng/mL), COC were washed in fertilization medium (Tyrode medium with 25 mM bicarbonate, 10 mM lactate, 1 mM pyruvate, 6 mg/mL fatty acid free BSA, 100 μg/mL heparin and 40 μg/mL gentamycin). They were transferred into four-well dishes (50 COC/250 μL fertilization medium/well). Motile spermatozoa, obtained by centrifugation of frozen/thawed semen on a discontinuous percoll (Pharmacia, Uppsala, Sweden) density gradient (45/90%), were diluted in fertilization medium (2.10^6 ^spermatozoa/mL). COC and spermatozoa (250 μL of the previous suspension/well) were incubated together for 18 h at 38.8°C in a humidified atmosphere with 5% CO_2 _in 95% air. Semen from the same bull was used throughout experiments. Day of fertilization was considered as Day 0.

At the end of the fertilization period, presumptive zygotes were completely decoronated by vortexing and washed in modified synthetic oviduct fluid (mSOF) [34] with 5% FBS (MP Biomedicals, Illkirch, France). Then, they were cultured in a microdrop of mSOF with 5% FBS (25 embryos/25 μL) under paraffin oil at 38.8°C for 8 days in a water-saturated atmosphere of 5% CO_2, _5% O_2 _and 90% N_2_.

Embryonic cleavage (stage of 5 to 8 cells) and blastocyst rates were determined 48 h and 8 days after IVF of COC, respectively.

### RNA extraction and RT-PCR

Total RNA was isolated from whole ovary, dissected large and small follicles, corpus luteum and adipose tissue (as positive control) on ice into TRI Reagent buffer as advised by the manufacturer (Sigma-Aldrich). Oocytes, cumulus and granulosa cells were lysed in the same buffer and then freeze-thawed three times by rapid incubation in liquid nitrogen followed by immersion in water bath at room temperature. RNA was quantified by measuring the absorbance at 260 nm. To eliminate genomic DNA contaminations, RNA was treated with the DNA-free kit (Ambion, Courtaboeuf, France) according to the manufacturer's instructions and stored at -80°C until use.

Reverse transcription was performed on 1 μg of total RNA or 10 oocytes in a 20 μL reaction mixture containing 1× RT Buffer (Promega, Charbonnieres, France), 10 IU/μL Reverse transcriptase (Promega), 2 mM deoxynucleotide triphosphate (Pharmacia, St Quentin Yvelines, France), 7.5 μg/mL Oligo(dT) 15 Primer (Promega), 1 UI/μL RNAse inhibitor (RNAsin; Promega) for 1 h at 37°C.

Specific primer pairs (Invitrogen, Fischer Scientific, Strasbourg, France) used to amplify parts of adiponectin, adiponectin Receptor 1 and 2 are detailed in Table 1.

**Table 1:** Oligonucleotide primer sequences for RT-PCR amplification.

Primer name	Primer Sequence	Accession number	Product size (bp)
**AdipoR1**			
Sense	5'-ACA GTG GAG CTG GCC GAG CT-3'	NM 001034055	550
Antisense	5'-GAA CAC CAC CTT CTC CTG GA-3'		
**AdipoR2**			
Sense	5'-AGG AAC GGT GAC AGT GGC GT-3'	NM 001040499	520
Antisense	5'-CTG TGT GGA ACA GCC ATG AG-3'		
**Adiponectin**			
Sense	5'-AGG ACA ACA TGG AAG ATC CC-3'	NM 174742	410
Antisense	5'-GTA GTA GAG TCC CGG AAT GT-3'		

PCR was performed on a 25 μL volume containing, 2.5 mM MgCl_2_, 2 mM deoxynucleotide triphosphate, 1 × Taq polymerase buffer (New England Biolabs Inc., Ozyme, Saint Quentin Yvelines, France), 2 μM each primer and 40 IU/mL Taq polymerase (New England Biolabs Inc.). Thirty five PCR cycles of the following thermal conditions were performed in a 2720 Thermal Cycler (Applied Biosystems, Courtaboeuf, France): denaturation at 94°C for 5 min, specific annealing at 94°C for 1 min/58°C for 1 min/72°C for 1 min and extension at 72°C for 7 min. PCR products were visualized on 1.5% agarose gel stained with ethidium bromide and were extracted using the EZNA™ Gel Extraction kit (OMEGA bio-tek, VWR International, Fontenay-sous-Bois, France) as described by the manufacturer's protocol and sequenced by Genome Express (Meylan, France). The obtained sequences were compared with known bovine sequences in Genbank at the NCBI using the Basic Local Alignment Search Tool (BLAST) program on line [35].

### Protein extraction and western blotting

Total proteins were extracted from whole ovary, dissected large and small follicles, oocytes, embryos, corpus luteum, fresh-isolated and primary-cultured granulosa cells, cumulus cells and adipose tissue (as positive control) on ice in lysis buffer (10 mM Tris (pH 7.4), 150 mM NaCl, 1 mM EDTA, 1 mM EGTA, 0.5% Igepal) containing protease inhibitors (2 mM PMSF, 10 mg/ml leupeptin) and phosphatase inhibitors (100 mM sodium fluoride, 10 mM sodium pyrophosphate, 2 mM sodium orthovanadate). Lysates were centrifuged at 16 000 g for 30 min at 4°C, and the protein concentration in the supernatants was determined using a colorimetric assay (kit BC Assay protein quantification; Interchim, Montluçon, France).

Protein extracts (80 μg per lane) were separated by electrophoresis on 12% (w:v) SDS-polyacrylamide gel and transferred to 0.45 μm nitrocellulose membrane (Bio Trace™ NT; Pall Corporation, VWR International). The membranes were then blocked with 5% nonfat dry milk powder (NFDMP) or 5% BSA in Tris-buffered saline (TBS: 2 mM Tris-HCl, 15 mM NaCl, pH 7.4), containing 0.1% Tween-20 (TBST) for 45 min at room temperature. The membranes were incubated overnight at 4°C with appropriate antibodies (dilution 1/1 000), in TBST with 5% NFDMP or 5% BSA. After several washes in TBST, they were incubated for 2 h at room temperature with a horseradish peroxidase-conjugated anti-rabbit or anti-mouse or anti-goat IgG (dilution 1/5 000) in TBST with 5% NFDMP. After washing again in TBST, the signal was detected by enhanced chemiluminescence (Western Lightning *Plus*-ECL, Perkin Elmer, Life and Analytical Sciences, Courtaboeuf, France). The specific bands were quantified with the software Scion Image for Windows (Scion Corporation, Maryland, USA). The results are expressed in arbitrary units as the signal intensity normalized to the signal for vinculin or total tested protein for phosphorylated protein.

### Immunohistochemistry

Ovarian biopsies were fixed for 12 h in 4% paraformaldehyde in PBS (pH 7.2). After serial dehydratation steps, the samples were embedded in paraffin and serially sectioned at a thickness of 7 μm. Sections were deparaffinized, hydrated, and were immersed in a hydrogen peroxide/methanol/water (0.3%/10%/87%; v/v/v) mixture for 20 min at room temperature to quench endogenous peroxidase activity. After two 5-min washes in PBS, sections were twice microwaved for 2 min in an antigen unmasking solution (Vector Laboratories, Inc., AbCys, Paris, France), left to cool to room temperature and washed in PBS for 5 min. Then nonspecific background was eliminated by blocking with 5% lamb serum in PBS for 30 min, followed by incubation overnight at 4°C with rabbit IgG (dilution 1/200, as negative control) or with rabbit primary antibody raised against either adiponectin (Acrp30 (N-20)-R, dilution 1/50), or adiponectin receptor 1 (2 μg/mL) or adiponectin receptor 2 (2 μg/mL) in PBS with 5% lamb serum. Sections were washed twice in PBS for 5 min and were incubated for 30 min at room temperature with a ready-to-use labeled polymer-HRP antirabbit antibody (Kit DakoCytomation EnVision Plus System-HRP; Dako, Trappes, France). After washing twice in PBS for 5 min, immunoreactivity was revealed by incubation of the sections in DAB chromagen/DAB buffer as described in the kit manual (Kit DakoCytomation EnVision Plus System-HRP) at room temperature. The slides were counterstained with hematoxylin, then dehydrated and mounted in Depex. Immuno-specific staining (brown) was observed using an Axioplan Zeiss transmission microscope coupled with a numerical camera piloted by the Software Spot (version 4.0.1 for Windows; Diagnostic Instruments, Inc, MicroMecanique, Evry, France).

### [^3^H] Thymidine incorporation into bovine granulosa cells

Granulosa cells (2 × 10^5 ^viable cells/400 μL medium/well) were cultured into 24-well dishes in enriched McCoy's 5A medium supplemented with 10% FBS and amphotericin B (2.7 μmol/L) for 24 h. After 24 h of serum starvation, the medium was removed and one μCi/mL of [^3^H] thymidine (Perkin Elmer; Life and Analytical Sciences, Courtaboeuf, France) was added in the presence or absence of adiponectin (10 μg/mL) and/or IGF-I (10^-8 ^M) and insulin (10^-8 ^M). After 24 h of culture, the radioactivity was determined in scintillation fluid by counting in a β-photomultiplier as previously described [36]. The values, expressed as counts per min (CPM), are representative of three independent cultures with each condition in triplicate.

### Progesterone and estradiol radioimmunoassay

Granulosa cells (1.25 × 10^5 ^viable cells/250 μL medium/well) were cultured in 48-well dishes. After serum starvation, the cells were incubated in the presence or absence of adiponectin (10 μg/mL) and/or IGF-I (10^-8 ^M) or insulin (10^-8 ^M) for 48 h. The concentration of progesterone and estradiol in the culture medium of bovine granulosa cells was measured by a radioimmunoassay protocol as previously described [37]. The results are expressed as the amount of steroids (ng/mL) secreted per 48 h per 50 μg protein and per basal amount. They are representative of four independent cultures with each condition in quintuplet.

The concentration of progesterone was also measured in the TCM199 serum-free culture medium after 24 h of IVM of COC (20 COC/200 μL medium/well) in the presence or absence of adiponectin (10 μg/mL) and/or insulin (10^-8 ^M). For COC, the results, representative of four independent experiments, are expressed as amount of progesterone (ng/mL) secreted per 24 h.

### Measure of oocyte nuclear maturation

Oocytes were denuded by mechanical separation of cumulus cells either immediately after collection (immature oocytes at germinal vesicle stage, 0 h of IVM) or after 24 h of IVM in TCM 199 serum-free medium with or without adiponectin (10 μg/mL) and/or insulin (10^-8 ^M) and/or EGF (10 ng/mL) + 10% FBS into four-well dishes (NUNC, Roskilde, Denmark). For each experimental condition, 20-50 oocytes per experiment were analyzed for their nuclear status. Oocytes were put on slides in a droplet of PBS/0.1%BSA, dried for 10 min and fixed in 96% ethanol overnight. Chromatin labelling was performed by incubation of slides in Hoechst33342 solution (1 mg/mL in 10 mM sodium citrate) for 1 min, followed by mounting of slides in Moviol. Observations were performed on fluorescent Axioplan Zeiss microscope. Results are expressed as a percentage of oocytes in metaphase-II stage.

### Statistical analysis

Results, expressed as mean ± SE relative or not to the basal state, are representative of at least three independent experiments. Statistical analyses were carried out using a factorial ANOVA test following by a Fisher's PLSD test, when the ANOVA revealed significant effects. Data were considered statistically significant at *P *< 0.05.

## Results

### mRNA and protein expression of adiponectin system in bovine ovary and embryo

**Figure 1:**
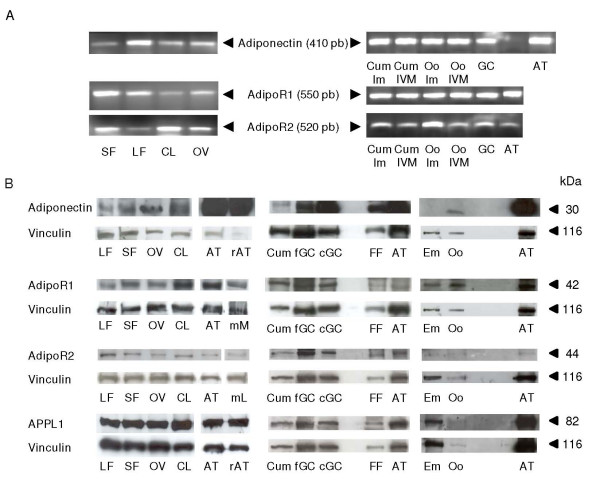
**Expression of adiponectin system (mRNA and proteins) in bovine ovary and embryo**. (A) RT-PCR analysis of the mRNAs for adiponectin, AdipoR1 and AdipoR2 in small (SF) and large (LF) follicles, corpus luteum (CL), whole ovary (Ov), cumulus cells from immature (Cum Im) and 24-h IVM (Cum IVM) COC, immature (Oo Im) and 24-h IVM (Oo IVM) oocytes, granulosa cells (GC) and adipose tissue (AT). (B) Detection of adiponectin (30 kDa), AdipoR1 (42 kDa), AdipoR2 (44 kDa) and APPL1 (82 kDa) by immunoblotting in LF, SF, OV, CL, AT, cumulus cells (Cum, from 40 24-h IVM COC per lane), fresh (fGC) and 48-h cultured (cGC) granulosa cells from small follicles (< 6 mm), follicular fluid (FF), oocytes (Oo, n = 100 per lane; except for APPL1, n = 20 per lane) and embryo (Em, *in vitro *day-8 blastocyst, n = 55 per lane). Rat adipose tissue (rAT) was used as positive control for the presence of adiponectin and APPL1, as already tested in our laboratory with the present antibodies. Mouse muscle (mM) and mouse liver (mL) were used as positive controls for the presence of AdipoR1 and AdipoR2 respectively, because these two antibodies were described to cross react with mouse. Vinculin protein was used as a loading control (n = 3).

RT-PCR analysis revealed the amplification of cDNAs corresponding to fragments of adiponectin (410 pb), AdipoR1 (550 pb), AdipoR2 (520 pb) in whole ovary, large and small follicles, corpus luteum, granulosa cells, cumulus cells from mature and immature COC and oocytes, as shown in adipose tissue used as a positive control (Fig. 1A). The sequence analysis of each fragment showed 100% homology with each Bos Taurus predicted sequence of adiponectin, AdipoR1 and Adipo R2, respectively (accession numbers in Table 1).

Adiponectin (as the monomeric form, 30 kDa), AdipoR1 (42 kDa), AdipoR2 (44 kDa) and APPL1 (82 kDa) were revealed by immunoblotting in whole ovary, large and small follicles, corpus luteum, cumulus cells, granulosa cells, follicular fluid and oocyte (Fig. 1B). Whereas adiponectin seemed to be less expressed in bovine fresh-isolated granulosa cells than in bovine primary-cultured granulosa cells (for 48 h) from small follicles (decrease by about 60%), the receptors AdipoR1 and AdipoR2 and the adaptor APPL1 were present at similar levels in the two cell types (Fig. 1B). In embryos (n= 55 blastocysts day 8/per lane), AdipoR1 and APLL1 were clearly expressed, while AdipoR2 and adiponectin were weakly present and undetectable, respectively (Fig. 1B).

**Figure 2:**
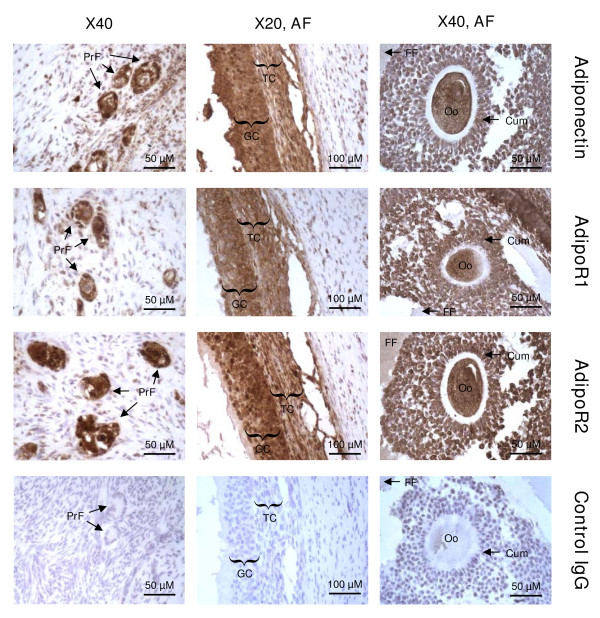
**Localization of adiponectin, AdipoR1 and AdipoR2 in bovine ovary by immunohistochemistry**. Adiponectin, AdipoR1 and AdipoR2 were detected in primary (PrF) and antral (AF) follicles, granulosa cells (GC), cumulus cells (Cum), theca cells (TC), oocyte (Oo), and follicular fluid (FF). Negative controls included a section incubated with rabbit IgG (n = 3).

The protein localization of adiponectin and its receptors (AdipoR1 and AdipoR2) was performed by immunohistochemistry. As shown in Fig. 2, adiponectin and its receptors were present in primary and antral follicles and more precisely in granulosa, cumulus and theca cells, oocyte and follicular fluid.

### Effect of rh adiponectin on bovine granulosa cell proliferation

**Figure 3:**
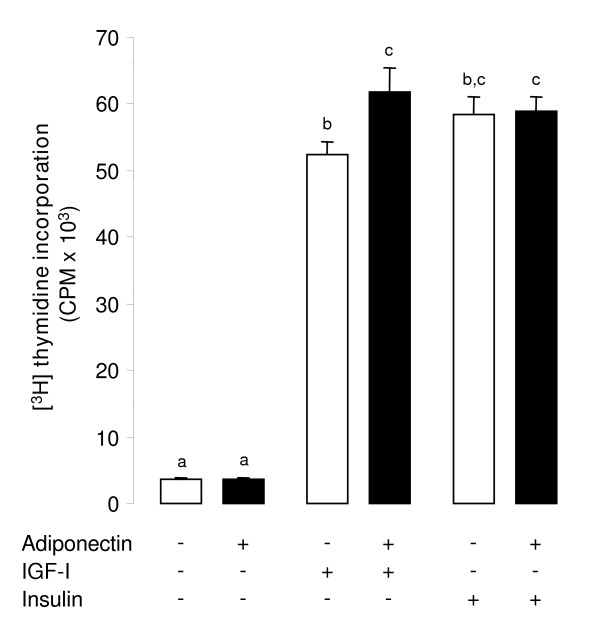
**Effect of rh adiponectin on proliferation of bovine granulosa cells**. [^3^H] thymidine incorporation was determined in bovine granulosa cells cultured for 24 h in enriched McCoy's 5A medium (without FBS) supplemented with or without rh adiponectin (10 μg/mL), ± IGF-I (10^-8 ^M), or insulin (10^-8 ^M). The data are expressed as mean ± SE and the measure unit of [^3^H] thymidine incorporation is counts per min (CPM). The results are representative of three independent cultures with each condition in triplicate. Bars with different superscripts are significantly different (*P *< 0.05).

We next examined whether the supplementation of rh adiponectin affected the proliferation of primary bovine granulosa cells (from small follicles, < 6 mm) by induction of mitosis. Therefore, we measured the [^3^H] thymidine incorporation in these cells after 24 h of culture in serum-free medium with or without rh adiponectin (10 μg/mL) in the presence or absence of recombinant human IGF-I (10^-8 ^M) or insulin (10^-8 ^M). As expected, IGF-I and insulin significantly increased [^3^H] thymidine incorporation in granulosa cells by 14- and 15-fold, respectively (*P *< 0.0001 for both; Fig. 3). Rh adiponectin did not modify the basal or insulin-induced cell proliferation, however it significantly increased (+18.2%) the proliferation of bovine granulosa cells stimulated by IGF-I (*P *= 0.004; Fig. 3).

### Effect of rh adiponectin on bovine granulosa cell steroidogenesis

**Figure 4:**
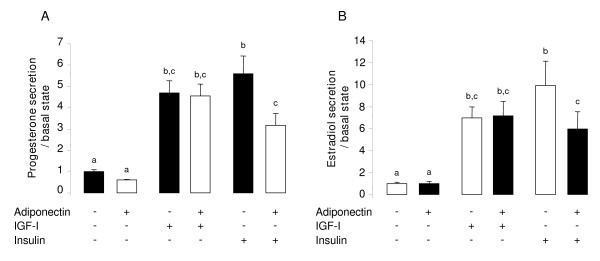
**Effect of rh adiponectin on steroidogenesis of bovine granulosa cells**. Progesterone (A) and estradiol (B) secretions were measured by RIA protocol in culture medium of granulosa cells after 48 h of culture in enriched McCoy's 5A medium (without FBS) with or without rh adiponectin (10 μg/mL), +/- IGF-I (10^-8 ^M), or insulin (10^-8 ^M). The data are expressed as the amount of steroids (ng/mL) secreted per 48 h per 50 μg protein and per basal amount. The results, expressed as means ± SE, are representative of four independent cultures with each condition in quintuplet. Bars with different superscripts are significantly different (*P *< 0.05).

Granulosa cells from small follicles (< 6 mm) were cultured 48 h in serum-free medium supplemented with or without rh adiponectin (10 μg/mL) in the presence or absence of IGF-I (10^-8 ^M) or insulin (10^-8 ^M). The progesterone and the estradiol productions were measured in the culture medium by RIA protocol. As shown in Fig. 4A, the progesterone secretion was significantly increased by IGF-1 and insulin treatments compared to the basal state, by 4.7- and 5.6-fold (*P *< 0.0001 for both) respectively. Likewise, we observed a significant 7- (*P *= 0.0097) and 10-fold (*P *< 0.0001) increase in the estradiol production (Fig. 4B), when the granulosa cells were cultured in the presence of IGF-I and insulin, respectively. No significant effect of rh adiponectin was observed on basal and IGF-I-induced secretions of steroids by primary bovine granulosa cells, whereas the insulin-induced productions of progesterone and estradiol were significantly decreased by 43.2% (*P *= 0.0026) and 39.8% (*P *= 0.0459), respectively.

### Effect of rh adiponectin on various signaling pathways in bovine granulosa cells

**Figure 5:**
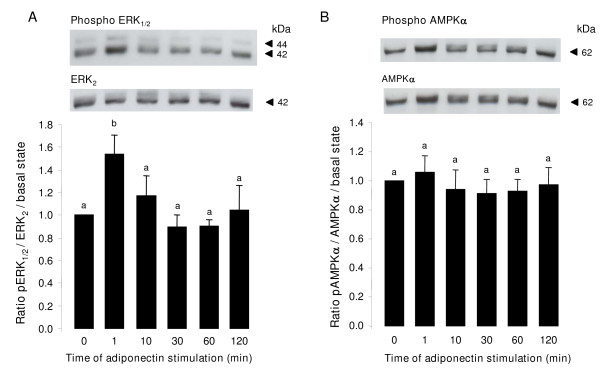
**Effect of rh adiponectin on phosphorylation of MAPK ERK_1/2 _(A) and AMPK (B) in bovine granulosa cells**. Granulosa cells from small follicles were cultured for 24 h in enriched McCoy's 5A medium supplemented with 10% FBS, starved of serum for 18 h and then incubated in serum-free medium with or without adiponectin (10 μg/mL) from 1 to 120 min. Protein extracts were separated by electrophoresis on 12% (w:v) SDS-polyacrylamide gel. After transfer to nitrocellulose membranes, the proteins were probed with anti-phospho-ERK_1/2 _or anti-phospho-AMPKα. The blots were stripped and reprobed with antibodies against ERK_2 _or AMPKα, respectively. Bands on the blots were quantified and the phosphorylated/total protein ratio was calculated. Values represent means ± SE relative to the basal state from at least three independent experiments. Bars with different superscripts are significantly different (*P *< 0.05).

We analyzed the pattern of ERK_1/2 _and p38 MAPK, AMPK and Akt phosphorylation in primary bovine granulosa cells following different times of rh adiponectin stimulation (10 μg/mL). As shown in Fig. 5A, we observed a significant rapid and transient increase of phosphorylated ERK_1/2 _MAPK (+ 53.8%) after 1 min of rh adiponectin treatment (*P *= 0.0094). Conversely, rh adiponectin had no effect on phosphorylation of AMPKα from 1 to 120 min in bovine granulosa cells (Fig. 5B). The AMPK signaling pathway was not modified by rh adiponectin after 5 min or 3, 4, 6, 12, 18 or 24 h of stimulation either (data not shown). We observed no significant effect of rh adiponectin treatment on phosphorylation of Akt and p38 MAPK in bovine granulosa cells from 1 to 120 min (data not shown).

### Effect of insulin on ERK_1/2 _MAPK signaling pathway in bovine granulosa cells stimulated with rh adiponectin

**Figure 6:**
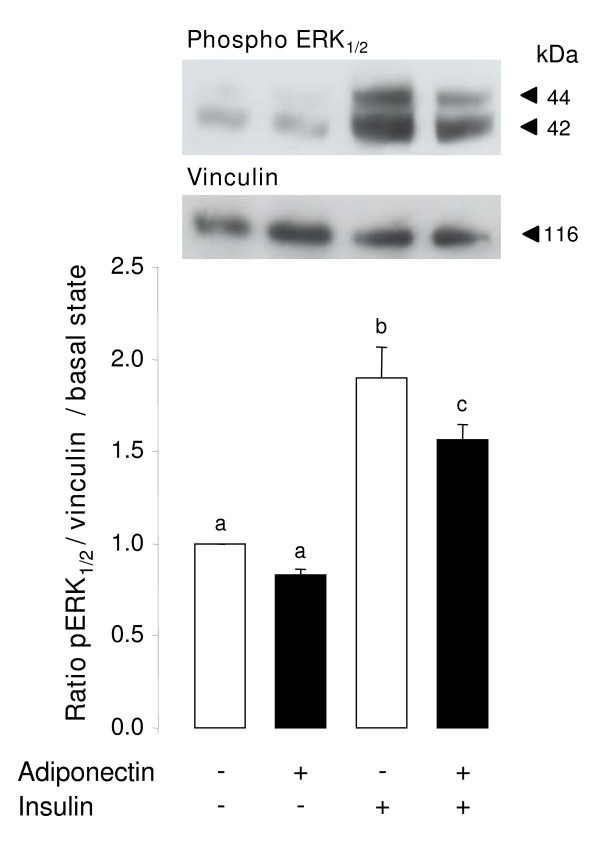
**Effect of insulin after 24-h stimulation with rh adiponectin on phosphorylation of MAPK ERK_1/2 _in bovine granulosa cells**. Granulosa cells from small follicles were cultured for 24 h in enriched McCoy's 5A medium supplemented with 10% FBS, starved of serum for 24 h and then incubated in serum-free medium with or without rh adiponectin (10 μg/mL) for 24 h. Then, granulosa cells were cultured in fresh serum-free medium with or without insulin (10^-8 ^M) for 5 min. Protein extracts were separated by electrophoresis on 12% (w:v) SDS-polyacrylamide gel. After transfer to nitrocellulose membranes, the proteins were probed with anti-phospho-ERK_1/2_. The blots were stripped and reprobed with antibodies against vinculin. Bands on the blots were quantified and the phospho-ERK_1/2_/vinculin ratio was calculated. Values represent means ± SE relative to the basal state from three independent experiments. Bars with different superscripts are significantly different (*P *< 0.05).

To investigate whether adiponectin decreases insulin-induced steroid production in bovine granulosa cells through a regulation of the ERK_1/2 _MAPK signaling pathway, primary granulosa cells were cultured in the presence of rh adiponectin (10 μg/mL) for 24 h followed by a 5-min stimulation with insulin (10^-8 ^M). As shown in Fig. 6, we observed a significant increase of ERK_1/2 _MAPK phosphorylation (+ 89.7%, *P *= 0.0001) in the presence of insulin. This induction was significantly decreased (-17.6%, *P *= 0.0351), when granulosa cells were 24-h pretreated with rh adiponectin (Fig. 6). Conversely, no significant effect of rh adiponectin was observed on Akt phosphorylation in granulosa cells in response to insulin (data not shown).

### Effect of rh adiponectin on bovine COC during in vitro maturation (IVM)

**Figure 7:**
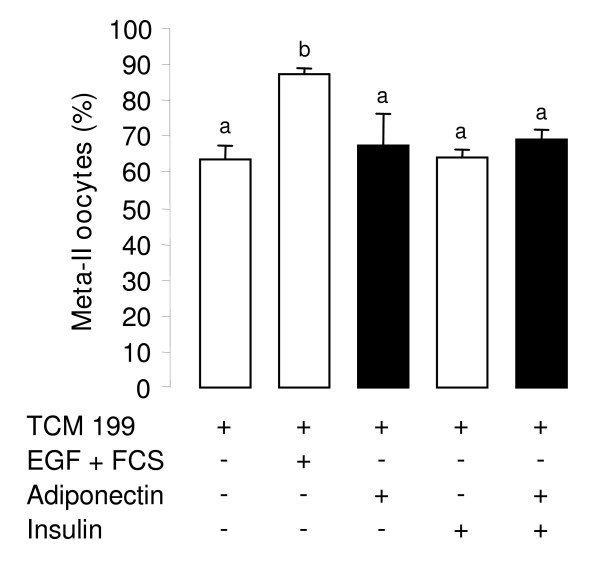
**Effect of rh adiponectin on 24-h nuclear maturation of bovine oocyte *in vitro***. *In vitro *maturation of COC was performed in TCM 199 serum-free medium (control) supplemented or not with EGF (10 ng/mL) + 10% FBS (positive control), or rh adiponectin (10 μg/mL) ± insulin (10^-8 ^M) for 24 h. The percentage of oocytes in metaphase-II stage was determined by chromatin labelling with Hoechst33342, as described in material and methods section. The data represents mean values ± SE from three independent experiments. Bars with different superscripts are significantly different (*P *< 0.05).

In order to study the effects of adiponectin on nuclear maturation of oocytes, COC were matured *in vitro *for 24 h in TCM 199 serum-free medium supplemented with or without EGF (10 ng/mL) + 10% FBS, ± rh adiponectin (10 μg/mL) ± insulin (10^-8 ^M). We examined the cumulus expansion and the percentage of matured oocytes (in metaphase-II stage). As expected, the EGF + FBS treatment increased the cumulus expansion (data not shown) and the percentage of matured oocytes *in vitro *(87.1% versus 63.7% for control, *P *= 0.0044) (Fig. 7). Conversely, rh adiponectin did not modify either basal or insulin-induced cumulus expansion after the IVM period (data not shown). Likewise, the percentage of nuclear-matured oocytes was not significantly changed by the rh adiponectin treatment independent of the presence of insulin (Fig. 7).

**Figure 8:**
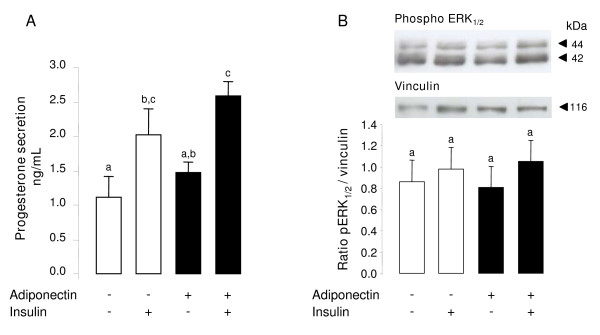
**Effect of rh adiponectin on progesterone secretion by bovine COC (A) and phosphorylation of MAPK ERK_1/2 _in these complexes (B)**. Progesterone secretion (A) was measured by RIA protocol in culture medium of COC (20 COC/200 μL medium/well) after 24 h of IVM in TCM 199 serum-free medium supplemented or not with rh adiponectin (10 μg/mL) +/- insulin (10^-8 ^M). The data are expressed as the amount of progesterone (ng/mL) secreted per 24 h. The results, expressed as means ± SE, are representative of four independent cultures. (B) IVM of COC (15 COC/well) was performed in TCM 199 serum-free medium supplemented or not with rh adiponectin (10 μg/mL) ± insulin (10^-8 ^M) for 1 h. Protein extracts were separated by electrophoresis on 12% (w:v) SDS-polyacrylamide gel. After transfer to nitrocellulose membranes, the proteins were probed with anti-phospho-ERK_1/2_. The blots were stripped and reprobed with antibodies against vinculin. Bands on the blots were quantified and the phospho-ERK_1/2_/vinculin ratio was calculated. Values represent means ± SE relative to the basal state from three independent experiments. Bars with different superscripts are significantly different (*P *< 0.05).

Progesterone produced by cumulus cells has been shown to play an important role on oocyte maturation [38,39] and rh adiponectin greatly decreased the insulin-induced progesterone secretion by bovine granulosa cells in the present work. Thus, we investigated the effect of rh adiponectin (10 μg/mL) on progesterone secretions by COC cultured for 24 h in TCM 199 serum-free medium ± rh insulin (10^-8 ^M). As expected, a 1.8-fold increase of progesterone secretion was observed in medium of COC supplemented with insulin versus medium of COC without treatment (*P *= 0.0388; Fig. 8A). Conversely, rh adiponectin had no significant effect on basal or insulin-induced progesterone production by COC after 24 h of IVM (Fig. 8A).

We next investigated the effect of adiponectin on the ERK_1/2 _MAPK pathway in COC matured *in vitro *for 1 h in TCM 199 serum-free medium with or without rh adiponectin (10 μg/mL) ± insulin (10^-8 ^M). In our conditions, no significant effect of rh adiponectin was observed on ERK_1/2 _MAPK phosphorylation in COC after 24-h IVM independent of the presence of insulin (Fig. 8B). Likewise, AMPK phosphorylation in COC and ERK_1/2 _MAPK phosphorylation in oocyte was not significantly modified by rh adiponectin treatment in the presence or absence of insulin (data not shown).

### Effect of rh adiponectin on bovine early embryo development

**Figure 9:**
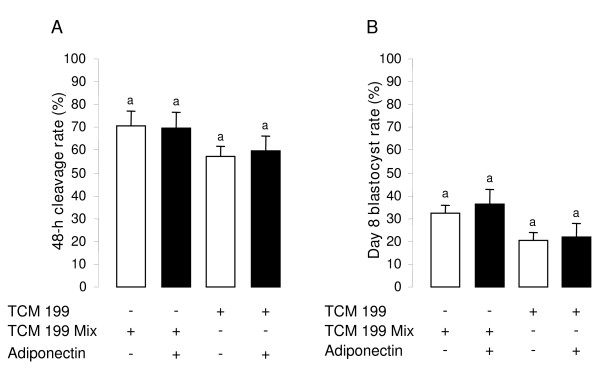
**Effect of rh adiponectin on bovine early embryo development *in vitro***. After 24 h of IVM in TCM 199 serum-free medium or TCM 199 mix with or without rh adiponectin (10 μg/mL), COC were *in vitro *fertilized in the specific IVF medium for 18 h. The zygotes were completely denuded of cumulus cells and cultured for 8 days in the IVD medium. We measured the cleavage rate (A) and the blastocyst rate (B) 48 h and 8 days after IVF, respectively. The results, expressed as mean ± SE, are representative of four independent experiments. Bars with same superscripts are not significantly different (*P *≥ 0.05).

We investigated the effect of rh adiponectin (10 μg/mL) supplemented during IVM (in TCM 199 mix or TCM 199 serum-free media) on 48-h cleavage and day 8 blastocyst rates after *in vitro *fertilization (IVF) and *in vitro *development (IVD) in bovine. We found, that rh adiponectin did not modify both the 48-h cleavage and day 8 blastocyst rates independent of the culture IVM medium (Fig. 9A and 9B). Moreover, we tested the effect of rh adiponectin on early embryo development, after 24-h IVM of bovine COC in TCM 199 serum-free medium enriched with EGF (10 ng/mL) with or without insulin (10^-8 ^M), and demonstrated no effect of rh adiponectin either on 48-h cleavage rate or blastocyst day 8 rate under these conditions (data not shown).

## Discussion

Here, we showed for the first time in cow that a 48-h treatment of rh adiponectin (10 μg/mL) greatly decreased insulin, but not IGF-1-induced steroid production in the culture medium of primary granulosa cells from small follicles, while a 24-h treatment slightly increased IGF-1-induced proliferation of these cells. In addition, we found a significant rapid and transient increase of phosphorylated ERK_1/2 _MAPK after 1 min of rh adiponectin stimulation in bovine granulosa cells, whereas the AMPK, Akt and p38 MAPK signaling pathways were not affected. A significant decrease in ERK_1/2 _MAPK phosphorylation was noticed after a 5-min stimulation of insulin (10^-8 ^M) in bovine granulosa cells pre-treated for 24 h with rh adiponectin (10 μg/mL). Finally, we did not observe significant effect of adiponectin (10 μg/mL) on bovine oocyte maturation and early embryo development *in vitro*.

In cow, the presence of adiponectin, AdipoR1 and AdipoR2 mRNA has been already described in theca and granulosa cells [22,32], but adiponectin, AdipoR1, AdipoR2 and APPL1 proteins have never been identified in ovary. Using RT-PCR, western-blotting and immunohistochemistry approaches, we found that adiponectin, AdipoR1 and AdipoR2 mRNA and proteins were present in whole bovine ovary and more particularly in small and large follicles, corpus luteum, oocyte, granulosa, theca and cumulus cells. Expression of adiponectin and its two receptors has already been reported in ovarian structures of several species, including pig [21,40], rat [2], chicken [15,41,42] and human [16]. Our results showed a great expression of adiponectin in granulosa cells, which could be a specificity of bovine species. Indeed, other studies on human or rat and chicken have observed no [16] or a weak [2,15] mRNA and protein expression of adiponectin in granulosa cells, respectively. AdipoR1 and AdipoR2 become biologically active by binding to a protein adaptor, called APPL1 [19]. As adiponectin and its receptors, APPL1 protein was present in all cell types of bovine follicles. Thus, our findings underline an *in situ *production of adiponectin and its two receptors in oocyte, theca, cumulus and granulosa cells, suggesting potential autocrine and/or paracrine effects of adiponectin on bovine ovarian cells.

Schmidt *et al*. found the presence of mRNA of the two adiponectin receptors in preimplantation mouse and rabbit embryos, whereas adiponectin mRNA was only detected in rabbit day 6, and not in mouse day 3.5 blastocysts [25]. In pig, Chappaz *et al*. reported the protein expression of AdipoR1 and AdipoR2 in blastocysts day 6 by immunolocalization [43]. Our western-blotting data showed that AdipoR1, AdipoR2 (less than AdipoR1) and APLL1 were expressed in bovine blastocysts day 8 following IVF and IVD, while we failed to detect adiponectin. This lack of adiponectin expression in bovine embryo could be due to a weak quantity of protein used in our immunoblot conditions (55 embryos per lane), or a too weak production of adiponectin during early embryo development, or to the fact that the protein analysis was performed on *in vitro *embryo devoid of maternal environment. Indeed, Schmidt *et al*. showed in mouse the absence of adiponectin mRNA in blastocysts day 3.5 prior to implantation but the presence of adiponectin protein in trophoblast cells of implanted embryos on day 5 post-coitum [25]. Adiponectin protein was also localized in mouse endometrial tissue of pregnant mice on day 3.5, suggesting a role of maternal and embryo exchanges for adiponectin expression in blastocyst. In cow, the protein expression of AdipoR1, AdipoR2 and APPL1 in blastocyst supports the idea of a direct action of adiponectin on the embryonic cells and a potential role of this adipokine in embryo development.

Our results suggest that adiponectin could affect some functions of bovine granulosa cells. Before studying steroidogenesis and proliferation, we checked by western-blotting that the whole adiponectin system remained expressed in primary culture of granulosa cells from small follicles. As already described for adiponectin and its receptors in rodent primary granulosa cells [2], the adiponectin system was still detected in bovine granulosa cells after several days of culture, while we observed an increase in adiponectin expression (about + 60%) in primary granulosa cells than in fresh ones. This increase could be linked to the luteinized status of primary granulosa cells in our conditions of culture.

The role of adiponectin on cell proliferation depends on the cell type. Indeed, this adipokine has been reported to inhibit the growth of various cancer cell lines [44,45], whereas it promotes mitosis of primary rat and human osteoblasts [46] and Human Embryonic Kidney 293 cells [20]. Here, we explored the potential involvement of rh adiponectin (10 μg/mL) on proliferation of bovine primary granulosa cells from small follicles in the presence or absence of IGF-1 (10^-8 ^M) or insulin (10^-8 ^M) for 24 h. As already described [47], IGF-1 and insulin significantly increase bovine granulosa cell proliferation. In agreement with results obtained in rat and human granulosa cells [2,16], we observed no effect of adiponectin on basal granulosa cell proliferation in cow. Likewise, insulin-induced proliferation of bovine granulosa cells was not modified by adiponectin treatment in our experiments. In bovine theca cells from large follicles, Lagaly *et al*. observed no effect of adiponectin (3 μg/mL) on cell proliferation in the presence of insulin and LH, as well [22]. In rat [2] and human [16], the effect of adiponectin (5 μg/mL) was also studied on granulosa cell proliferation in the presence of IGF-1, but adiponectin treatment had no effect. Conversely in cow, we revealed a weak but significant increase of IGF-1-induced cell proliferation by 18% in response to adiponectin in granulosa cells from small follicles. The discrepancy between the results could be due to the species or the different doses of adiponectin used in the experiments.

We also investigated the effects of rh adiponectin (10 μg/mL) on steroidogenesis of bovine primary granulosa cells from small follicles in the presence or absence of IGF-1 (10^-8 ^M) or insulin (10^-8 ^M) for 48 h. As already reported in cow [36,47] and other species [2,15,48], we found that progesterone and estradiol productions were stimulated by IGF-1 and insulin treatments in the culture medium of bovine granulosa cells. In our study, no significant effect of adiponectin was observed on basal secretions of progesterone and estradiol by bovine granulosa cells. This lack of adiponectin effect on basal steroidogenesis is not specific to the bovine species or to the adiponectin concentration used in experiments, since it has been previously observed in rat with 5 μg/mL of adiponectin [2], in chicken with a 10 μg/mL dose [15] and in human with a 5 μg/mL concentration [16]. Nevertheless, Ledoux *et al*. have shown that adiponectin (25 μg/mL) was able to increase the steroidogenic acute regulatory protein (StAR) mRNA abundance and decrease those of CYP19A1 in porcine granulosa cells [40]. However, in this latter study, the authors did not examine the direct effect of adiponectin on progesterone and estradiol secretions. Our group reported a stimulation of IGF-1-induced progesterone and (or) estradiol productions by adiponectin in the culture medium of granulosa cells in human [16], hen [15] and rat [2]. However in cow, we demonstrated here no effect of adiponectin on progesterone and estradiol secretions induced by IGF-1 in granulosa cells from small follicles. Similarly, Lagaly *et al*. showed that adiponectin did not affect the mRNA expression of two key enzymes of steroidogenesis, CYP19A1 and CYP11A1, in bovine granulosa cells from large follicles treated with IGF-1 (30 ng/mL) and FSH (30 ng/mL) [22]. Conversely, we found that adiponectin greatly decreased both progesterone and estradiol productions by about 40% in response to insulin in the culture medium of bovine granulosa cells. Our results are in good agreement with those observed in bovine theca cells from large follicles, where adiponectin was able to decrease progesterone and androstenedione in the culture medium supplemented with insulin (100 ng/mL) and LH (100 ng/mL) [22]. Thus, adiponectin may inhibit steroidogenesis induced by insulin in both granulosa and theca cells in bovine species.

Several groups have already described a positive effect of adiponectin on several signaling pathways, including MAPK, AMPK and Akt in various cell types [3,49]. In the present study, we investigated the effects of rh adiponectin (10 μg/mL) on the phosphorylation of ERK_1/2 _and p38 MAPK, AMPKα and Akt in bovine granulosa cells from 1 to 120 min. Curiously, we observed no significant modification of AMPKα phosphorylation from 1 to 120 min but also after 5 min or 3, 4, 6, 12, 18 and 24 h of treatment with adiponectin, whereas recombinant adiponectin stimulated AMPK in porcine, rat and chicken granulosa cells [2,15,40]. However, a recent study in human also revealed that adiponectin (5 μg/mL) did not activate AMPK in granulosa cells [16]. Adiponectin rapidly and transiently activated ERK_1/2 _MAPK in bovine granulosa cells, as previously described in porcine, rat, chicken and human granulosa cells [2,15,16,40]. The ERK_1/2 _MAPK and/or Akt signaling pathways have been potentially involved in the steroid production in rat [50,51] and bovine [52,53] granulosa cells. To understand through which pathway adiponectin decreased insulin-induced steroidogenesis in bovine granulosa cells, we analyzed the pattern of ERK_1/2 _MAPK and Akt phosphorylation in bovine granulosa cells cultured with rh adiponectin (10 μg/mL) for 24 h and then with insulin (10^-8 ^M) for 5 min. We observed a significant decline of insulin-induced ERK_1/2 _MAPK, but not Akt phosphorylation in bovine granulosa cells pre-treated with adiponectin. Thus, the decrease in insulin-induced ERK1/2 phosphorylation in response to adiponectin could contribute to explain the reduction in insulin-induced steroid production in cultured bovine granulosa cells, but additional experiments using specific ERK1/2 inhibitors for example are needed to confirm this hypothesis.

In the present work, we demonstrated the presence of the adiponectin system in bovine cumulus cells, oocyte, and blastocyst (except for adiponectin) suggesting a potential involvement of adiponectin in bovine oocyte maturation and embryo development. Here, rh adiponectin (10 μg/mL) in IVM medium for 24 h affected neither the cumulus expansion and the meiotic maturation (percentage of metaphase-II oocytes) of bovine oocytes derived from small follicles, nor the 48-h cleavage and blastocyst day 8 rates. Conversely, Chappaz *et al*. have found in pig, that adiponectin (30 μg/mL) decreased the proportion of immature oocytes from large follicles at 46 h of IVM, suggesting an acceleration of the meiotic maturation of porcine oocyte by adiponectin [43]. This discrepancy between their results and ours could be linked to animal species or to the size of the follicles used for COC isolation. Indeed, Chappaz *et al*. observed no effect of adiponectin on meiotic maturation of porcine oocyte from small follicles [43]. This suggests that oocyte derived from small follicles may require further *in vivo *maturation to respond efficiently to adiponectin stimulation. Finally, Chappaz *et al*. did not find any modification of the cleavage rate after IVM of parthenogenetically activated porcine oocytes in the presence of recombinant adiponectin (30 μg/mL) [43]. Conversely, they observed an improvement of the blastocyst rate when activated porcine oocytes were matured *in vitro *without adiponectin and then embryo were developed for 7 days in the presence of adiponectin [43].

## Conclusions

We identified adiponectin, AdipoR1, AdipoR2 and APPL1 in bovine granulosa, theca cells, corpus luteum, oocyte, and cumulus cells. Interestingly, adiponectin was strongly expressed in bovine granulosa cells. In bovine embryo, we also demonstrated the presence of AdipoR1, AdipoR2 and APPL1. Furthermore, we showed that rh adiponectin decreased insulin but not IGF-1-induced progesterone and estradiol secretions by bovine granulosa cells and this was associated with a partial inhibition of the ERK_1/2 _MAPK phosphorylation. Rh adiponectin stimulated proliferation of bovine granulosa cells induced by IGF-1 but not by insulin. Finally, in our conditions bovine oocyte maturation and *in vitro *early embryo development are not modified by rh adiponectin. The present findings provide new elements towards understanding the role of adiponectin within the bovine ovary. However, further investigations are required to elucidate the mechanisms involved in different effects of adiponectin on steroid secretions and cell proliferation of cultured ovarian cells and in whole animal to understand the potential role of adiponectin in dairy cow fertility.

## Competing interests

The authors declare that they have no competing interests.

## Authors' contributions

VM, SU, FG and CP participated together with JD in the design of the study. The experiments were carried out by VM, SU, FG, CP, CR, SCC. Data analysis was performed by VM. The manuscript was written by VM. All authors read and approved the final manuscript.
